# Alcoholism Treatment in the United States

**Published:** 1994

**Authors:** Mary E. McCaul, Janice Furst

**Affiliations:** Mary E. McCaul, Ph.D., is an associate professor and Janice Furst, Ph.D., is an instructor in the Department of Psychiatry and Behavioral Sciences, Johns Hopkins University School of Medicine, Baltimore, Maryland

## Abstract

Almost 600,000 patients are treated for alcohol abuse and alcohol dependence in the United States each year. Yet no one treatment approach has been shown to be successful for all these patients. Innovative treatment modalities are being studied in an effort to make alcoholism treatment more effective and more economical.

It is estimated that 9.6 percent of men and 3.2 percent of women in the United States will become alcohol dependent at some time in their lives (Grant 1992); many more men and women will exhibit drinking behavior that can be classified as alcohol abuse. Alcohol dependence is a chronic, primary psychiatric disorder characterized by a cluster of recognizable symptoms, including alcohol tolerance (i.e., needing more alcohol to become intoxicated); physical withdrawal; loss of control over drinking; and continued use of alcohol despite social, medical, family, or occupational problems (American Psychiatric Association [APA] 1994). Alcohol abuse is less severe than alcohol dependence. It is characterized by harmful consequences of drinking (e.g., failure to fulfill major social, family, or vocational obligations; recurrent alcohol use in physically dangerous situations; and repetitive legal problems) but without the development of alcohol tolerance, physical withdrawal, or compulsive alcohol use (APA 1994).

Both alcohol dependence and alcohol abuse are disorders that can and should be treated. According to a 1991 survey of alcoholism and other drug abuse treatment facilities and their clients, almost 575,000 clients were treated in 8,928 facilities in the United States (U.S. Department of Health and Human Services [USDHHS] 1993). Of these clients, 12 percent were admitted to inpatient programs, and 88 percent were treated as outpatients. Sixty-seven percent of the clients were white; 17 percent were African-American; 12 percent were Hispanic; and 4 percent were Asian, Native American, and other ethnic groups. About 60 percent of the clients were in their twenties and thirties, 6 percent were adolescents under 18, and 5 percent were over age 55. Twenty-five percent of the clients in treatment were women.

This overview describes current types and innovative components of alcoholism treatment and evidence of their effectiveness. In addition, treatment needs of priority populations,^1^ including older persons, women, and minority patients, are discussed. Current interest in patient-treatment matching as a means to increase the individualization of services and, consequently, their effectiveness are considered. Finally, cost-effectiveness and financing trends are addressed briefly.

## Treatment Settings

Alcoholism treatment services are delivered in two general settings—inpatient and outpatient. Inpatient settings mostly consist of short-term residential programs. They often are used for the early phases of treatment, particularly acute detoxification. Outpatient settings provide more long-term maintenance treatment, with group meetings and individual counseling offered once or twice a week. Because of current concern over increasing health care costs, more emphasis now is being placed on outpatient care during all stages of recovery. As a result, successful models of outpatient detoxification and intensive day treatment services have been developed.

### Inpatient Treatment

Residential 28-day treatment programs traditionally have been considered the foundation of the early recovery period. These programs often are based on the disease model of alcoholism and on the 12-step or Alcoholics Anonymous (AA) philosophy and practices. Abstinence from alcohol and other drugs (AOD’s) is the primary treatment goal in these programs. Patients participate in frequent AA meetings and ideally are linked to an AA sponsor and a local AA chapter prior to discharge.

Inpatient settings, such as hospitals, provide intensive, highly structured treatment, such as group therapy and alcoholism education, for several hours daily. Issues covered in alcoholism education include health consequences, course of the disease, effects on the family, and other relevant topics. In addition to these group activities, clients work individually with a counselor to develop and implement a treatment plan that defines the treatment goals and to receive personalized therapy for special problems or needs. An essential element of residential programs appears to be milieu treatment, that is, living with a large number of alcoholic patients who have had similar experiences and problems and who can offer insight and advice on the recovery process. Professional staff are available during the treatment’s early stages to manage medical problems and to conduct a psychosocial evaluation to guide the treatment process. Toward the end of the hospitalization, treatment often involves the client’s family, and the spouse or other family members may be asked to join the treatment process.

Walsh and colleagues (1991) recently investigated the effectiveness of inpatient alcoholism treatment. Alcoholics who were identified through employee assistance programs were entered randomly into one of three treatment options: compulsory residential treatment followed by AA attendance, compulsory community-based AA attendance alone, or patient choice of treatment modality. The majority of patients in the third group chose either inpatient treatment (41 percent) or AA attendance (46 percent). All three groups showed substantial and fairly stable improvement in alcohol consumption and employment status over the 2-year followup period; patients in inpatient programs, however, improved most on several measures of AOD use. Although these results suggest the effectiveness of residential treatment, they cannot be generalized, because no formal outpatient treatment group was included in the study design.

A recent study by Finney and Moos (1991) also supports favorable long-term outcomes for alcoholics following residential treatment. Ten years after treatment, married or cohabiting alcoholics were compared with a community sample matched on various health, demographic, and psychosocial characteristics. About 70 percent of the patients were abstinent or stable nonproblem drinkers during the last 5 years, and only 30 percent had relapsed to heavy drinking. Recovered patients appeared equivalent to the community sample on measures of mental and physical health and occupational and family functioning, whereas relapsed patients generally fared worse than the community sample. These findings underscore that many alcoholics are treated successfully and have a favorable long-term treatment outcome.

In recent years, inpatient treatment programs have undergone substantial changes. For example, length of stay in many programs has decreased dramatically as a result of increased emphasis on outpatient interventions and of cost pressures from the insurance industry. Whether this affects treatment outcome has not been determined.

The client population also has changed in recent years and now includes more multiple substance abusers than before. Consequently, the emphasis in many programs has shifted from a focus on alcoholism only to a focus on combined AOD dependence.

### Outpatient Treatment

The majority of alcoholic patients are treated on an outpatient basis. In 1991, 88 percent of the clients who sought treatment for alcohol problems were treated in outpatient facilities (USDHHS 1993). These facilities offered detoxification services (to about 3,200 clients), intensive outpatient care (to about 52,400 clients), and regular outpatient services (to about 641,400 clients).

#### Intensive Outpatient Care

Intensive outpatient programs were modeled after psychiatric day treatment programs, which emerged in the 1970’s as alternatives to inpatient hospitalization. The intensive outpatient programs vary considerably in the amount of time that patients are treated, ranging from 8 hours per day, 7 days per week, to 3 hours per day, several days per week. Several well-controlled studies comparing inpatient and intensive outpatient treatment have demonstrated comparable long-term outcomes but significantly lower costs in the intensive outpatient setting. For example, Fink and colleagues (1985) reported improved rates of abstinence from alcohol and improved mood for patients treated in an intensive outpatient program compared with those in an inpatient program. Comparable outcomes for the two groups were found for other measures of alcohol involvement, job stability, and interpersonal status (e.g., functioning in parental and spousal roles). Longabaugh and colleagues (1983) demonstrated that the treatment costs were much lower in intensive outpatient than in inpatient treatment.

Intensive outpatient treatment may offer two kinds of advantages. First, it has clinical advantages by allowing patients to practice relapse prevention and management skills while being in a highly structured treatment setting. Second, it has practical advantages, such as the ability to serve larger numbers of patients; increased scheduling flexibility (e.g., offering evening programs for employed patients); and an opportunity for the patients to maintain their established roles of employee, spouse, and/or parent while receiving intensive treatment.

#### Regular Outpatient Care

Regular outpatient alcoholism services are used as primary treatment or as extended aftercare following completion of an inpatient or intensive outpatient program. These types of outpatient services usually include weekly group therapy sessions, regular individual counseling sessions with an alcoholism counselor, participation in AA meetings, and family therapy when appropriate. The recommended treatment length generally is at least 1 year. The number of treatment sessions and the length of stay in outpatient treatment are related positively to long-term improvement in drinking behavior and other psychosocial areas (Polich et al. 1980).

Outpatient programs can support and enhance the improvements achieved in inpatient treatment. In a study by McLatchie and Lomp (1988), residential patients were assigned randomly to groups with no, with voluntary, or with mandatory outpatient aftercare participation for 12 weeks. Clients who completed the outpatient program had substantially lower relapse rates than noncompleters, regardless of the specific number of after-care sessions or type of aftercare requirement. Similarly, Gilbert (1988) found that patients who completed an aftercare program reported more abstinent days and fewer alcohol dependence symptoms than patients who dropped out. These studies highlight the role of patient compliance as a determinant of treatment outcome and suggest that greater emphasis should be placed on strategies that encourage patients to follow the treatment program.

### Self-Help Programs

Since the 1940’s community-based self-help programs have grown considerably. They are now widely available and offer an important intervention resource for people with alcohol problems. The best-known and most frequently used self-help program is AA. AA groups are self-governed and independent of formal alcoholism treatment facilities. Meetings are conducted by recovering alcoholics, without regard to formal counseling training and experience. Critical elements of the AA program include fellowship meetings, with members expected to attend 90 meetings in 90 days during the early recovery period; a sponsor system, in which newly recovering alcoholics are linked to an established member for assistance and advice; and the 12-step philosophy. This philosophy spells out a series of activities or steps that the alcoholic should achieve in the recovery process.

It has been difficult to study AA’s effectiveness as a treatment intervention, in part because of the anonymity of AA members and in part because of the inability to conduct randomized clinical trials in an AA setting. One recent study found AA participation to be the only significant predictor of length of sobriety during long-term followup (Cross et al. 1990). Being an AA sponsor correlated particularly strongly with sobriety: 91 percent of the sponsors reported complete or stable recovery. Although such analyses are confounded by the fact that AA participants may be more motivated for recovery than are other patients, these findings suggest that participation in self-help programs can assist in the alcoholism recovery process and should be studied further (for more information, see the article by Tonigan and Hiller-Sturmhöfel, pp. 308–310).

## Treatment Components

Often the first step of alcoholism treatment is managing the effects of abrupt cessation of alcohol intake through pharmacologically assisted detoxification. The majority of treatment components, however, strive to achieve long-term abstinence or reduced alcohol consumption in the patients. Recently, there has been increased emphasis on evaluating focused, standardized therapeutic interventions, which may be offered alone or as part of larger treatment programs. Components that show particular promise for effective treatment fall into two broad categories. One category consists of strategies to reduce alcohol use directly, such as brief interventions, pharmacotherapy, behavioral self-control training,^2^ and cue extinction.^3^ The second category includes strategies to reduce alcohol use indirectly through enhancement of social and coping skills (e.g., behavioral marital therapy, relapse prevention, and stress management) (Holder et al. 1991; Miller 1992; for more information on behavioral treatment, see the article by Kadden, pp. 279–286).

### Detoxification

Alcohol withdrawal can be a potentially life-threatening medical problem requiring medical care; however, fewer than 10 percent of alcohol-dependent patients are at risk for severe withdrawal symptoms. Thus, the challenge for cost-effective withdrawal management is to identify those patients who need pharmacological support and intensive medical care in an inpatient setting.

Benzodiazepine medications (e.g., Valium^®^, Ativan^®^) are used widely for alcohol withdrawal management. Some investigators support benzodiazepine treatment for all patients with moderate-to-severe withdrawal symptoms because of possible long-term, cumulative central nervous system damage associated with repeated unmedicated alcohol withdrawal episodes, even if each episode seems innocuous (Linnoila et al. 1987). Other investigators have attempted to develop standardized approaches for identifying patients in need of medication and for determining drug dosage levels. For example, Wartenberg and colleagues (1990) used the revised Clinical Institute Alcohol Withdrawal Scale to monitor symptom severity among patients in an inpatient detoxification unit. They found that only 13 percent of the patients needed medication, whereas 73 percent of the patients were medicated before this assessment tool was used. Although fewer patients were medicated, there was no increase in the overall incidence of withdrawal complications or early termination of treatment. Patients could benefit from decreased benzodiazepine use by experiencing less sedation and drowsiness, which may interfere with short-term rehabilitation therapy. In addition, benzodiazepines have an abuse potential of their own, and their extensive use may cause behavioral problems in patients requesting larger doses than are recommended.

Because the management of alcohol withdrawal with medications is safe for most patients, outpatient detoxification models have been developed. Hayashida and colleagues (1989) compared benzodiazepine-assisted withdrawal for alcoholics in both an outpatient and an inpatient setting. Although more patients dropped out of outpatient detoxification, no differences between the two groups were found in withdrawal complications and transfer rates into long-term rehabilitation. Six months after detoxification, patients in both groups reported comparable improvements in alcohol consumption and psychosocial status.

### Brief Interventions

Growing evidence indicates the effectiveness of brief interventions in decreasing alcohol use by heavy drinkers who are not yet physically dependent on alcohol (Institute of Medicine [IOM] 1990). Physicians and other health care professionals deliver these interventions based on an assessment of the patient’s alcohol use and alcohol-related problems. During a brief, highly directive consultation, the professional informs the patient about the assessment results (e.g., elevated liver functions, absenteeism or lateness at work, or alcohol-related arrests). The professional then offers clear strategies, such as goal setting and contracting, to reduce future drinking.

Researchers have studied the effectiveness of brief interventions in inpatient and outpatient health care settings. The findings indicate that brief interventions reduce alcohol use and improve health status when compared with no intervention (Wallace et al. 1988) and can be as effective as more extended treatment protocols (Chick et al. 1988). Bibliotherapy, a type of brief intervention in which patients receive written materials on the harmful effects of alcohol and guidelines for reducing drinking, also has been found to reduce alcohol consumption and associated problems (Sanchez-Craig et al. 1989). These interventions represent a potentially powerful and cost-effective tool for early treatment of heavy drinkers identified in a variety of settings.

### Pharmacotherapy

Three types of medications have received the most attention in research conducted on the long-term treatment of alcohol abuse/dependence: alcohol-sensitizing drugs, anticraving drugs, and drugs that treat concurrent psychiatric disorders (Litten and Allen 1991; for more information, see the article by Anton, pp. 265–271).

#### Alcohol-Sensitizing Medications

Such alcohol-sensitizing medications as Antabuse^®^ are used to discourage patients from drinking during their rehabilitation program. When combined with alcohol, these drugs produce unpleasant effects, including facial flushing, nausea, vomiting, and increased blood pressure and heart rate. In a comprehensive well-controlled study of the effectiveness of Antabuse, Fuller and colleagues (1986) reported that the medication did not improve abstinence rates, the length of time to relapse, or psychosocial functioning more than did counseling alone. Patients on Antabuse who did not remain abstinent, however, drank less frequently than relapsed patients who did not receive the medication.

For almost all patients, Antabuse appears to act through a psychological mechanism rather than through the actual pharmacological interaction with alcohol; that is, the patients believe that they will become sick if alcohol is ingested and, therefore, do not drink (Allen and Litten 1992). Thus, patients who comply with the administration protocol typically achieve successful outcomes without actually experiencing the alcohol-sensitizing reaction. As a result, increased attention has been given to strategies to improve compliance with the recommended medication regimen (Allen and Litten 1992).

#### Anticraving Medications

Over the last several decades, research has shown that various neurochemicals (i.e., chemical messengers that modulate responses of neurons in the brain) play a role in the development of alcohol consumption, tolerance, and dependence. Studies now are focusing on neuroregulating medications to reduce craving for alcohol and alcohol’s rewarding or intoxicating effects. One potential target is the opioid regulatory system. Two recent studies have reported that naltrexone, which blocks opiate receptor sites in the brain, decreases alcohol consumption and relapse in alcohol-dependent men enrolled in outpatient alcoholism treatment (O’Malley et al. 1992; Volpicelli et al. 1992; also see the article by Volpicelli et al., pp. 272–278).

#### Psychiatric Medications

Many alcoholic patients report high levels of anxiety and depression when entering treatment. As a result, many studies have examined the effectiveness of a variety of antianxiety and antidepressant medications in both clinically anxious or depressed alcoholics and in patients who do not meet the clinical definitions for these psychiatric disorders (Liskow and Goodwin 1987; Litten and Allen 1991). The effectiveness of these drugs is still controversial. Many earlier studies did not find significant reductions in drinking as a consequence of pharmacological intervention (Dorus et al. 1989; Liskow and Goodwin 1987). In addition, safety concerns for some of these medications have been raised. These concerns include potential abuse of some psychiatric medications, dangers associated with mixing alcohol and medications, altered metabolism and elimination of some medications because of chronic alcohol use, and the risk of noncompliance with the therapy by patients with comorbid disorders (Zweben and Smith 1989).

More recently, researchers have studied medications that reduce the uptake of the neurochemical serotonin (e.g., fluoxetine [Prozac^®^] or zimelidine) and nonbenzodiazepine antianxiety drugs, such as buspirone. These drugs have known antidepressant properties and also decrease food and fluid consumption; either mechanism could decrease alcohol use. Naranjo and colleagues (1990) found a 20- to 25-percent reduction in alcohol consumption in heavy drinkers who received Prozac compared with patients who did not receive the medication.

### Marital/Family Therapy

Reviews of marital and family therapy in alcoholism treatment have supported the importance of involving family members in the treatment process (IOM 1990). Spouse involvement improves both marital and alcohol use outcomes during the early posttreatment period (McCrady et al. 1986). Because of the difficulty of engaging spouses and other family members in long-term therapy, brief marital/couples therapy has been studied as a cost-effective alternative to more traditional extended counseling. Zweben and colleagues (1988) found that a single session of advice counseling for couples improved drinking status and marital satisfaction to the same extent as eight sessions of couples therapy.

McCrady and colleagues (1991) studied the effectiveness of three levels of couples therapy: minimal therapy, therapy focusing on alcohol effects only, and alcohol/behavioral marital therapy (ABMT) that addressed alcohol-related problems and general marital communication skills. Patients in all three groups had similar reductions in drinking 6 months after treatment. After 18 months, however, the number of abstinent days increased for patients who received ABMT and decreased for patients in the other two groups. ABMT patients also reported improved marital satisfaction and lower rates of marital separations than the other patients. Thus, improved general marital communication skills may be beneficial for both decreasing alcohol use and increasing marital stability. These benefits may become more evident after long-term followup as the patients master and integrate communication skills into their marital interactions.

### Relapse Prevention

Relapse during the recovery process can be triggered by a variety of intrapersonal and interpersonal factors. Intrapersonal cues include stress, depression, and levels of alcohol craving and withdrawal. Interpersonal factors include the negative life events and daily inconveniences that an individual experiences, as well as interpersonal tension. Relapse prevention strategies have been developed to teach alcoholics how to cope effectively with potential relapse triggers.

Monti and colleagues (1990) found that providing patients with training in both mood management (e.g., awareness and management of anger and negative moods) and communication skills (e.g., assertiveness, starting conversations, nonverbal communication, or receiving criticism) reduced alcohol use and improved psychosocial adjustment. Yet not all patients respond to all treatments equally. Mood management training is more effective for patients who have less education and who have high levels of anxiety and craving (Rohsenow et al. 1991). The effectiveness of communication skills training, in contrast, is not influenced by these pretreatment patient characteristics. These findings suggest that both approaches to relapse prevention can be effective but that intrapersonal mood states may be more difficult to change in some patient groups.

## Treatment for Priority Populations

Priority populations are defined as groups currently underserved in treatment programs or as groups requiring special interventions because of unique treatment needs and/or the relative ineffectiveness of standard treatment programs (IOM 1990). These groups include older persons, women, minorities, and adolescents (for information on the treatment needs of adolescents, see the article by Bukstein, pp. 296–301). Existing studies of treatment outcome for priority populations often failed to apply rigorous methodology in evaluating specialized services for these patient populations.

### Older Persons

The rates of alcohol abuse and alcohol dependence are lower among older individuals than among other age groups. Patients over age 55 compose about 5 percent of alcoholism treatment program admissions (USDHHS 1993). Although most older alcoholics report relatively stable heavy drinking throughout their life, about 40 percent of them experience a recent onset of alcohol problems (Hurt et al. 1988). This late-onset alcoholism is believed to result from aging-specific life stressors, such as retirement, physical illness, or death of a spouse.

**Figure:**
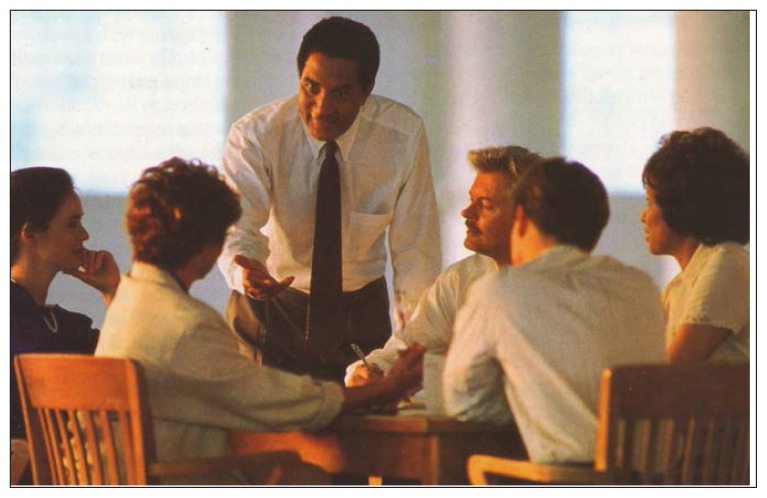
Treatment services that address specific needs and emphasize peer group participation can improve treatment outcomes for many patients.

Whether older patients need specialized treatment still is controversial. Most studies have found that older alcoholics do as well as younger alcoholics in mixed-age programs (Atkinson et al. 1985). Other investigators have compared treatment outcome in age-specific and mixed-age programs. Kofoed and colleagues (1987) found superior outcomes for older alcoholics who once a week attended a specialized group that used a slow pace, was less confrontational, and emphasized socialization and support. Patients in the specialized group remained in treatment longer, attended more group sessions, and were more than four times as likely to complete the program as were patients in mixed-age groups. Relapse rates for patients in both groups were similar, but relapse was treated more successfully for patients in the specialized group. These findings suggest that age may be an important matching variable when optimizing treatment.

### Women

Alcohol-abusing or alcohol-dependent women and men differ in a variety of biopsychosocial variables. For example, women typically begin drinking at a later age and seek treatment after a shorter duration of heavy drinking than men, suggesting a more rapid development of alcohol-related problems (Blume 1986). Compared with men, alcohol-abusing women also are at increased risk for depression, low self-esteem, alcohol-related physical problems, marital discord or divorce, spouses with alcohol problems, a history of sexual abuse, and a pattern of drinking in response to life crises (IOM 1990).

Although many of these gender differences could be important in designing effective alcoholism treatment for women, little information is available on the relative effectiveness of traditional mixed-gender programs and specialized treatment services. Dahlgren and Willander (1989) conducted a well-controlled study comparing women-only and traditional mixed-gender treatment. In both programs, the women received comparable individual and group counseling, occupational therapy, and medical care, but services in the specialized program focused specifically on women’s problems. Women in the specialized program remained in treatment longer, had higher completion rates, and had improved psychosocial and health outcomes compared with women in the mixed-gender program. These results indicate that treatment outcome for women may be better in specialized programs.

### Minorities

Some minority populations may be at increased risk for developing AOD dependence, with more rapid and/or severe medical and psychosocial consequences. For example, Hispanic men in detoxification were more severely dependent and showed greater cognitive impairment than did white or African-American men (Castaneda and Galanter 1988). Such differences, as well as language and cultural differences, suggest that providers of alcoholism treatment programs that serve minority clients should attempt to tailor their treatment curricula to meet the special needs of these clients. One strategy is to match therapists and patients on the basis of race or ethnicity (Nagy 1994). Westermeyer (1984) found that ethnically sensitive treatment programs were more successful in attracting minority patients. Clearly, more research is needed to identify the specialized needs of minority alcoholics and the extent to which treatment services targeted to minority clients can improve treatment outcome.

## Patient-Treatment Matching

Early reviews of alcoholism treatment generally concluded that although alcoholism treatment was more beneficial than no treatment, there was little evidence for a differential effectiveness of particular treatment approaches (e.g., Emrick 1975). This was attributable in part to the heterogeneity of patients and treatment approaches studied. The importance of patient-treatment matching research (i.e., analysis of the interactions between patient characteristics and type of treatment intervention) is becoming increasingly apparent.

A study by Kadden and colleagues (1989) illustrates the rationale for patient-treatment matching. The investigators examined the effectiveness of coping skills training and interactional group therapy designed to explore interpersonal relationships for patients with different levels of global psychopathology,^4^ so-ciopathy,^5^ and cognitive impairment. The study found that patients with higher levels of psychopathology and sociopathy had better outcomes with coping skills training, whereas patients with lower levels of psychopathology and sociopathy or with cognitive impairments did better with interactional therapy.

If it were possible to predict which treatments would be most beneficial for specific patient subgroups, it also would be possible to optimize the use of treatment resources, maximize treatment benefits for individual patients, and target treatment development for those patients not yet served effectively. To improve the understanding of patient-treatment matching, a multisite study entitled Project MATCH, supported by the National Institute on Alcohol Abuse and Alcoholism, currently is investigating the interactions of a range of patient variables with treatment outcome in three behavioral treatment approaches. (For more information on patient-treatment matching and on Project MATCH, see the article by Mattson, pp. 287–295.)

Several strategies already are being used in clinical practice that attempt to match patients to existing treatment modalities more effectively. For example, Hoffman and colleagues (1991) developed comprehensive guidelines for determining appropriate treatment placement using patient characteristics, such as psychosocial functioning, alcohol dependence severity, medical and psychiatric status, acute intoxication and withdrawal symptoms, and prior treatment and relapse history. Treatment characteristics used for matching include setting, staffing patterns, types of therapies, and ancillary support systems. Such structured guidelines can be useful for health care providers to validate treatment placement decisions, for insurance companies to monitor patient placement, and for researchers to evaluate treatment (IOM 1990). These strategies, however, have not yet been validated by experimental studies.

## Financing Trends in Alcoholism Treatment

Two research areas focus on the costs of alcoholism treatment. First, analyses of the cost-effectiveness of treatment compare costs and cost savings for treatment versus no treatment or among different treatment approaches. Second, studies of how alcoholism treatment should be financed compare public with private treatment settings and public with private insurance reimbursement systems.

### Cost-Effectiveness

Research consistently has shown that alcoholism treatment reduces overall medical care costs of alcohol-dependent clients. One recent study demonstrated that over a 14-year followup period, health care costs for treated alcoholics were 24 percent lower than for untreated alcoholics (Holder and Blose 1992). Only a few studies, however, have analyzed the relative cost-effectiveness of different types of treatment in different settings. Hayashida and colleagues (1989) compared alcohol detoxification in inpatient and outpatient settings. The study found no long-term differences in the effectiveness of inpatient and outpatient detoxification but found that costs were approximately 10 times higher in the inpatient than in the outpatient setting. Outpatient detoxification, therefore, may be a highly cost-effective alternative to traditional inpatient detoxification for patients who do not require immediate hospitalization.

Holder and colleagues (1991) reviewed the cost-effectiveness of interventions in 33 alcoholism treatment settings and treatment modalities. Treatments were rated on effectiveness (based on drinking outcomes in controlled studies) and cost (based on the recommendations of researchers conducting controlled trials for the least expensive setting and the minimum treatment duration). The study found that the more effective modalities consistently were in the minimal to medium-low cost range, whereas modalities with poor evidence of effectiveness generally were associated with higher costs. The investigators stress, however, that these results cannot be generalized because patient-treatment matching was not considered in their analysis. For example, although costly modalities may be rather ineffective for most patients, they may be necessary and cost-effective for specific high-need patient populations.

### Treatment Financing

Decisions regarding treatment setting are influenced strongly by pragmatic insurance coverage issues in addition to patient choice and treatment matching. For example, almost twice as many employees in insurance plans with more extensive inpatient coverage received inpatient care compared with employees whose plans had limited inpatient coverage (Holder and Blose 1991). Such findings underscore the need for a reexamination of current reimbursement strategies that often provide better coverage for inpatient or hospital-based services than for outpatient or nonhospital-based services.

A study of national trends in alcoholism treatment during the late 1970’s and early 1980’s (Yahr 1988) suggests that two separate alcoholism treatment systems are developing in this country—a private system for insured, financially stable patients and a public system for disadvantaged patients. According to the study, privately or corporately owned treatment facilities intended to generate profits typically offered medical detoxification and care in hospital settings and attracted suburban patient populations (i.e., the most economically advantaged residential category). Programs run by State or local governments or by nonprofit agencies, in contrast, offered more outpatient detoxification and care in outpatient or nonhospital facilities and served more inner-city and rural patient populations. During the study period, treatment capacities increased in the for-profit facilities but decreased slightly in nonprofit and State or local government programs. It will be important in future research to examine factors that may impact on these patterns, including the overall cost and effectiveness of different treatment components, the relative cost of treatment delivery by public- and private-sector units, and the correspondence between insurance coverage and use patterns of alcoholism treatment programs.

## Conclusions

The treatment of alcohol abuse and alcohol dependence has benefited from a variety of scientific and clinical advancements in the last decade. There is growing evidence for the effectiveness of outpatient settings for the delivery of treatment services for alcoholics in all stages of recovery. New focused treatment interventions have been identified that are effective and that can be offered alone or as part of more comprehensive treatment programs. For example, pharmacotherapy and marital skills training show promise for decreasing alcohol use and relapse risk.

Research on alcohol-dependent priority populations continues to highlight the unique problems and treatment needs of older persons, women, and minorities. Several studies provide encouraging evidence that treatment services addressing specific needs and emphasizing peer group participation can improve treatment outcomes for many patients. These findings lend further support to the importance of patient-treatment matching. An understanding of the interactions between specific patient, counselor, and treatment characteristics will yield the most successful long-term outcomes for all alcoholics. This information also will be critical in developing cost-effective financing and utilization management of alcoholism treatment in this country.

## Footnotes

1 For a definition of this term, see the section “Treatment for Priority Populations” on p. 257.

2 Behavioral self-control training consists of techniques or strategies intended to enable clients to manage or control their drinking using either abstinence or moderation as treatment goals.

3 In this approach, clients frequently are exposed to cues related to drinking (e.g., sight or smell of alcohol) without actually receiving alcohol. This reduces the physiological and psychological effects of the cues and thus reduces their effectiveness as triggers of alcohol use and relapse.

4 Global psychopathology included factors such as mental health problems, current and lifetime anxiety and depression, and suicidal thoughts or actions.

5 Measures of sociopathy examined the client’s level of conformity with societal norms (e.g., illegal activities).
